# ﻿Comparisons of insect and pathogen leaf damage on early Eocene *Eucalyptus* (Myrtaceae) from Patagonia and extant Australasian gum trees

**DOI:** 10.3897/phytokeys.266.166635

**Published:** 2025-11-17

**Authors:** L. Alejandro Giraldo

**Affiliations:** 1 Department of Geosciences and Earth and Environmental Systems Institute, Pennsylvania State University, University Park, PA 16802, USA Pennsylvania State University State College United States of America

**Keywords:** Agromyzidae, Australia, Gondwana, Gracillariidae, host specialization, host tracking, leaf mining, Nepticulidae

## Abstract

Australian forests and woodlands are dominated by the species-rich (> 700 spp.) genus *Eucalyptus* L’Hér. (Myrtaceae). Despite this modern-day dominance, the earliest macrofossil evidence of the genus comes not from Australia, but from the early Eocene Laguna del Hunco locality in Argentinean Patagonia, consisting of abundant vegetative and reproductive material. The leaves, assigned to the fossil species *Eucalyptus frenguelliana* Gandolfo & Zamaloa, record a diverse suite of insect and pathogenic damage that closely matches that observed on 36 extant, rainforest-associated *Eucalyptus* species. Here, I provide detailed morphological descriptions and photographic documentation of this damage, recorded on 284 *E. frenguelliana* leaves, together with extensive comparisons to analogous damage observed in extant *Eucalyptus* herbarium specimens (> 10,000 sheets reviewed). From the fossil material, I describe a diverse suite of 33 insect-mediated and pathogenic damage types (DTs), including twelve types of external feeding interactions, one of piercing-and-sucking marks, five of galls, ten of mines, three of pathogenic traces, and two of oviposition scars. This elevated number of DTs, encompassing a wide range of ecological interactions, indicates that *E. frenguelliana* was an important ecological resource in ancient Patagonian rainforests. Some of the fossil mines were probably created by micromoths in the families Nepticulidae and Gracillariidae, as well as flies in the family Agromyzidae. However, most of the insect and pathogenic damage observed in the fossils and their corresponding extant analogs was produced by still-unknown culprits, underscoring gaps in our knowledge of *Eucalyptus*-associated communities and their assembly through evolutionary time.

## ﻿Introduction

The gum-tree genus *Eucalyptus* L’Hér. (Myrtaceae) is often referred to as the “universal Australian” (Pryor and Johnson 1981) due to its dominance across the country’s forests and woodlands (Florence 1985; Ohmart and Edwards 1991). Today, the genus comprises over 700 species (Slee et al. 2020; Crisp et al. 2024), primarily native to Australia, with some naturally occurring in Indonesia, East Timor, Papua New Guinea, and the Philippines (Pryor and Johnson 1981; Williams and Woinarski 1997; Ladiges et al. 2003; Hill et al. 2016; Thornhill et al. 2019). Despite its ecological prominence in modern-day ecosystems, the Australian fossil record of *Eucalyptus* is scarce and often ambiguous, particularly for macrofossils (for reviews, see Rozefelds 1996; Hermsen et al. 2012; Hill et al. 2016; Macphail and Thornhill 2016; Rozefelds et al. 2024). Remarkably, the oldest macrofossil evidence of *Eucalyptus* comes not from Australia, but from the floristically diverse early Eocene (52 Ma) Laguna del Hunco (LH) locality in Argentinean Patagonia (Wilf et al. 2003; Gandolfo et al. 2011). The fossil material is confidently placed within crown group *Eucalyptus* based on taxonomic and phylogenetic analysis; diagnostic morphological characters for the genus seen in the fossils include falcate leaves with eucamptodromous venation, strong intramarginal veins, island oil glands, operculate flower buds, multistaminate flowers with in-situ *Myrtaceidites eucalyptoides* Cookson & Pike pollen, and pedunculate umbellasters bearing valvate capsulate fruits with a staminophore scar (Gandolfo et al. 2011; Hermsen et al. 2012; Zamaloa et al. 2020).

In addition to *Eucalyptus*, the nearest living relatives of many plant lineages described from LH today inhabit tropical rainforests of Australasia and Southeast Asia (Wilf et al. 2003, 2013, 2014, 2019, 2023, 2024; Zamaloa et al. 2006, 2020; Gandolfo et al. 2011; Wilf 2012, 2025; Carvalho et al. 2013; Kooyman et al. 2014; Rossetto‐Harris et al. 2020; Matel et al. 2022; Andruchow-Colombo et al. 2023). This consistent biogeographic pattern is the result of formerly widespread rainforests across southern Gondwana (South America, Antarctica, Australia), which were nearly exterminated once the final breakup of the supercontinent initiated in the early Eocene, triggering the loss of suitable habitat (Lagabrielle et al. 2009; Wilf et al. 2013; Kooyman et al. 2014; Dunn et al. 2015). However, several plant lineages survived in Australia and are still present there today or reached more distant lands in Australasia and Asia after the late Oligocene collision of the Sahul (Australia) and Sunda (Southeast Asia) plates (Hall et al. 2011; Wilf et al. 2013; Kooyman et al. 2014, 2019).

The *Eucalyptus* foliage from LH, assigned to the fossil species *Eucalyptus frenguelliana* Gandolfo & Zamaloa (Hermsen et al. 2012), has rich feeding traces, indicating that a diverse array of insect herbivores fed on this plant host (Wilf et al. 2005a; Carvalho et al. 2014; Giraldo et al. 2025). Previous work identified 28 insect herbivore damage types (DTs) on these fossils, all of which occur on extant, rainforest-associated *Eucalyptus* species, which probably reflects host-tracking through geologic time (Giraldo et al. 2025). The prior work provided selected illustrations of the fossil and comparable extant DTs, with limited morphological descriptions.

Here, I provide complete documentation of the biogeographically significant leaf damage observed in 284 fossil *E. frenguelliana* leaves from LH. I illustrate and provide detailed morphological descriptions for the 28 insect herbivory DTs previously discovered in Giraldo et al. (2025), including twelve pertaining to external feeding, one to piercing-and-sucking marks, five to galling, and ten to mining; as well as newly reported pathogenic traces. I also illustrate two types of previously published oviposition ichnotaxa (Sarzetti et al. 2009; Romero-Lebrón et al. 2019) for completeness (see Results). I discuss the potential affinities of the insect culprits responsible for the damage observed in the fossils, with a focus on leaf mining, and present more detailed comparisons with traces observed on extant *Eucalyptus* herbarium specimens. Some photographs from the prior work (Giraldo et al. 2025) are retained here to provide the reader with complete coverage of the damage suite in a single document.

## ﻿Methods

### ﻿Geological and environmental setting

All material examined in this study originated from the early Eocene caldera-lake deposits at Laguna del Hunco (LH), exposed near 42.5°S, 70°W in the Huitrera Formation, northwestern Chubut Province, Patagonia, Argentina (Wilf et al. 2003, 2005b; see Hajek et al. 2025 for updated maps and stratigraphy). Although LH preserves a diverse fossil biota, including insects and vertebrates (Fidalgo and Smith 1987; Báez and Trueb 1997; Azpelicueta and Cione 2011; Petrulevičius 2018; Degrange et al. 2021), its deposits are world-renowned for their exquisitely preserved and highly diverse plant material, including delicate structures such as flowers, fruits, and nearly complete infructescences (e.g., Zamaloa et al. 2006, 2020; Gandolfo et al. 2011; Wilf et al. 2017, 2023; Deanna et al. 2020; Matel et al. 2022; Wilf 2025).

The ~170 m of fossiliferous lake deposits at LH belong to the Middle Chubut River Volcanic Pyroclastic Complex (Aragón and Mazzoni 1997), and they consist mainly of tuffaceous mudstones and sandstones interbedded with airfall tuffs (Wilf et al. 2003, 2005b; Gosses et al. 2021; Hajek et al. 2025). LH lies within the 25–30 km wide Piedra Parada Caldera system, sitting stratigraphically above the Ignimbrita Barda Colorada, which formed the caldera floor (Aragón and Mazzoni 1997; Gosses et al. 2021). The stratigraphy and geochronology of the LH section was recently updated by Hajek et al. (2025), who found, according to U-Pb dating of three tuff layers, that nearly all fossils (and all studied here) were deposited between 52.217 ± 0.014 Ma and 51.998 ± 0.035 Ma (Hajek et al. 2025). Most fossils, including the *E. frenguelliana* leaves analyzed here, were recovered from facies associations B and C of Hajek et al. (2025), representing lake-floor environments in which most of the sediment was delivered via turbidity currents (facies B), or a mixture of hemipelagic sedimentation with intermittent wave energy, ashfall, and infrequent density flows (facies C).

These facies interpretations suggest that *Eucalyptus* and other plant remains at LH were transported downslope into the caldera lake by landslide-triggered density flows from the steep, vegetated caldera rim (Hajek et al. 2025). *Eucalyptus* trees probably grew on patches of barren soil after landslide and igneous disturbance occurred (Gandolfo et al. 2011; Wilf and Kooyman 2025), similar to the ecology of some extant non-Australian *Eucalyptus* species like *E. deglupta* Blume, which colonize landslide and lava-affected areas (Paijmans 1973; Adam 1994); or to Australian species such as *E. grandis* W.Hill ex Maiden and *E. pilularis* Sm., which occupy narrow bands along fire-disturbed rainforest margins (Harrington and Sanderson 1994; Tng et al. 2012b, 2012a; Wilf and Kooyman 2025).

### ﻿Fossil repository and photography

As detailed earlier (Giraldo et al. 2025), all fossil specimens are curated at the Museo Paleontológico Egidio Feruglio (MEF, repository acronym MPEF-Pb), Trelew, Chubut Province, Argentina. The fossil material, consisting of 284 *Eucalyptus frenguelliana* leaf specimens, was collected in multiple field expeditions to LH since 1999 (Wilf et al. 2003, 2005b; Gandolfo et al. 2011; Hermsen et al. 2012). A total of 277 *E. frenguelliana* leaf specimens are derived from 18 individual quarries, and seven additional specimens were found in float (see Suppl. material 1: dataset S1). These specimens represent all *E. frenguelliana* leaves observed in the field to have insect damage, including collections from early field censuses (Wilf et al. 2005b).

Fossil material was photographed or re-photographed at MEF using a DSLR Nikon D700 camera or a Nikon Eclipse 50i compound microscope with a Nikon DSFi3 camera and DS-L4 tablet controller. In cases where the surface of the fossils was uneven, photographs were composited to obtain high sharpness and depth of field. This was done using manual z-stacking and the Adobe Photoshop v.24.1 align and blend functions (for vertical stacking) and the Photomerge function (for lateral stitching). Reversible whole-image adjustments for white balance, temperature, and contrast were made using Adobe Camera Raw v.15.1.1. A high-resolution image library of the *E. frenguelliana* collection was previously made available in Giraldo et al. (2025) and can be publicly accessed in FigShare (doi: 10.6084/m9.figshare.24756975). Some photographs from the work of Giraldo et al. (2025) are reused here for completeness in documentation.

### ﻿Damage type scoring and analog damage on extant *Eucalyptus*

As described by Giraldo et al. (2025), each fossil leaf was inspected for insect-mediated and pathogenic damage at MEF using a Nikon SMZ1000 binocular microscope and scored for the presence of damage types (DTs) using customized keywords attached to images in Adobe Bridge (Rossetto-Harris et al. 2022), following the guide of Labandeira et al. (2007) and recent updates. Minor revisions here resulted in negligible changes in raw abundances for two DTs (DT33 and DT81), and the re-designation of DT85 (see fig. 3.bbof Giraldo et al. 2025) as DT215 (see Results and Suppl. material 1: datasets S1, S2).

I previously examined over 10,000 herbarium specimens from 36 extant, rainforest-associated *Eucalyptus* species in search of leaf damage comparable to that found in the fossil material (see Dataset S3 of Giraldo et al. 2025). Because such a high number of specimens was reviewed, collection bias against damaged specimens is likely to have been minimized (Meineke and Daru 2021). Although selected analog damage was illustrated in Giraldo et al. (2025), here I provide complete photographic documentation and detailed comparisons of the analog damage observed on the extant *Eucalyptus* specimens. To standardize nomenclature, I followed the most recent phylogeny of *Eucalyptus* (Crisp et al. 2024), and defined rainforest-associated *Eucalyptus* as those species whose ranges include (but are not necessarily limited to) the narrow fringes of rainforests (Harrington and Sanderson 1994; Tng et al. 2012b). These taxa were chosen because their ecological preferences coincide with the paleoenvironmental interpretation at LH, wherein *Eucalyptus* probably colonized disturbed areas adjacent to intact rainforests (Gandolfo et al. 2011; Wilf and Kooyman 2025; see Geological and environmental setting section above). Because Giraldo et al. (2025) were specifically testing for host-tracking in rainforest environments, revision of non-rainforest taxa is outside the scope of this work.

The herbarium review included ca. 7,400 herbarium sheets examined in person at **A**, **GH**, **CANB**, **CBG**, and **BRI**, and an additional ca. 3,100 high-resolution digital sheets from multiple herbaria, including **NSW**, **MEL**, **L**, **U**, **WAG**, **AMD**, **E**, **K**, **P**, **US**, and **NY** (institutional codes from Index Herbariorum; Thiers 2025). Digitized specimens were accessed primarily through the Australasian Virtual Herbarium and respective institutional portals, as detailed in Giraldo et al. (2025). Photographs of specimens taken in-person were captured using a Nikon D700 DSLR with a 105 mm f/2.8D lens, polarizing filter, and Nikon SB-R200 mini-flashes mounted on a mini-tripod.

## ﻿Results

**Figure 1.**
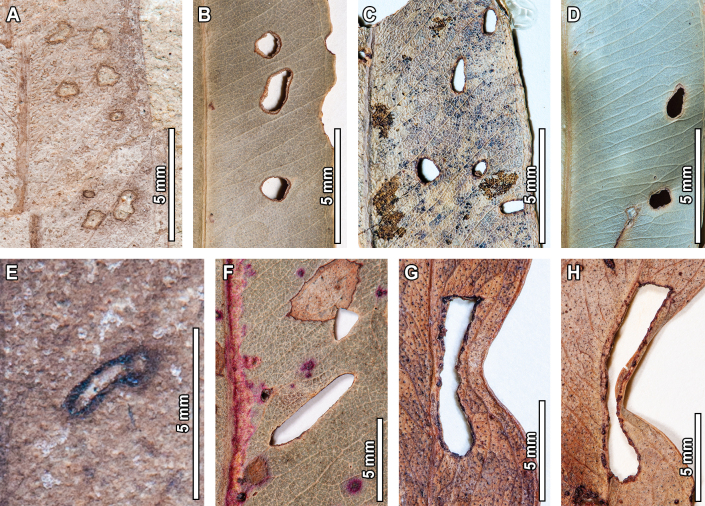
Hole feeding in fossil *Eucalyptus frenguelliana* leaves from Laguna del Hunco (A, E) and corresponding analogs in extant *Eucalyptus* species (B–D, F–H). A–D. Small circular (DT1) and polylobate (DT3) holes along the leaf lamina (A. MPEF-Pb 2314; B. *E. resinifera* J.White CANB [416146]; C. *E. saligna* Sm. A [*M.S.Clemens 1945*, no barcode]; D. *E. robusta* Sm. A [*J.L.Boorman 77155*, no barcode]); E–H. Elongate holes (DT8) with thick reaction tissue (E. MPEF-Pb 2245; F. *E. major* (Maiden) Blakely CANB [668871.1]; G. *E. nobilis* L.A.S.Johnson & K.D.Hill CANB [406091]; H. *E. nobilis* CANB [406086]).

**Figure 2.**
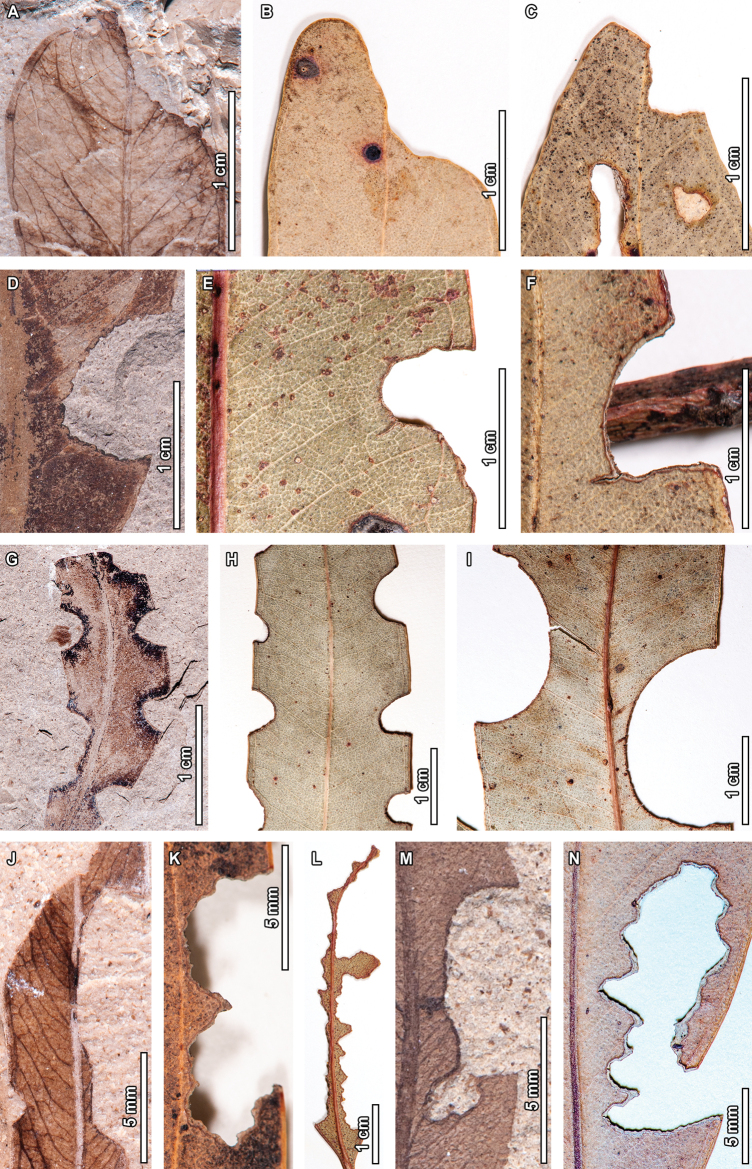
Margin feeding in fossil *Eucalyptus frenguelliana* leaves from Laguna del Hunco (A, D, G, J, M), and corresponding analogs in extant *Eucalyptus* species (B, C, E, F, H, I, K, L–N). A–C. Excisions removing leaf apices (DT13; A. MPEF-Pb 13030; B. *E. crebra* BRI [AQ0446860]; C. *E. microcorys* F.Muell. CANB [472647.1]); D–F. Semicircular excisions into the leaf margin (DT12; D. MPEF-Pb 13031; E. *E. michaeliana* CANB [435259]; F. *E. resinifera* CANB [891861.2]); G–I. Consecutive, nearly perfect semicircular excisions along the leaf margin (DT81; G. MPEF-Pb 13033; H. *E. michaeliana* CANB [435263], I. *E. grandis* CANB [699345]); J–L. Deep excisions reaching the midvein (DT14), similarly positioned along the leaf margin, with extreme examples (L) wherein almost all of the leaf tissue has been consumed (J. MPEF-Pb 8145; K. *E. siderophloia* Benth. BRI [AQ0146076], L. *E. nobilis* CANB [406105]); M, N. Deeply trenched excisions expanding towards the midvein (DT15; M. MPEF-Pb 2322; N. *E. fibrosa* F.Muell. A [*L.A.S.Johnson 61233*, no barcode]).

**Figure 3.**
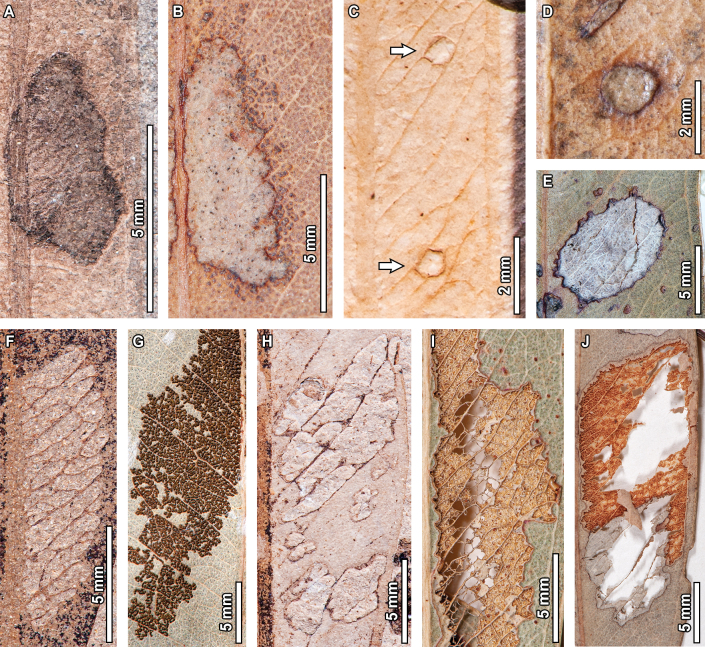
Surface feeding (A–E) and skeletonization (F–J) in fossil *Eucalyptus frenguelliana* leaves from Laguna del Hunco (A, C, F, H) and corresponding analogs in extant *Eucalyptus* species (B, D, E, G, I, J). A, B. Polylobate surface abrasions with clearly defined reaction rims (DT30), similarly positioned beside the midvein (A. MPEF-Pb 2250; B. *E. fibrosa* CANB [409185]); C–E. Circular surface abrasions with thick reaction rims (DT31; C. MPEF-Pb 2261; D. *E. robusta* CANB [15881]; E. *E. tereticornis* Sm. A [*R.Pullen 7221*, no barcode]); F, G. Skeletonized areas lacking a reaction rim (DT16) occurring alongside the midvein (F. MPEF-Pb 13034; G. *E. michaeliana* CANB [435213]); H–J. Skeletonized areas with a thick reaction rim (DT17) and similar patterns of breached tissue (H. MPEF-Pb 2327; I. *E. tereticornis* CANB [120052.1]; J. *E. crebra* BRI [AQ0098408]).

**Figure 4.**
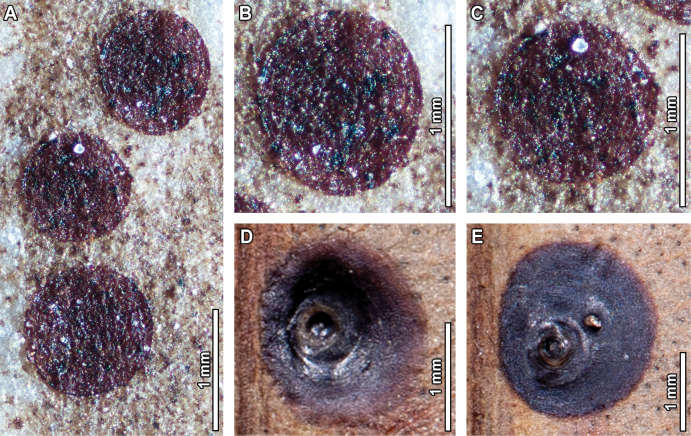
Piercing-and-sucking in fossil *Eucalyptus frenguelliana* leaves from Laguna del Hunco (A–C) and corresponding analogs in extant *Eucalyptus* species (D, E). A–E. Circular scale insect covers occurring in clusters (DT77; A–C. MPEF-Pb 2362; D, E. *E. notabilis* CBG [295.1]). Covers in (B) and (C) correspond to the upper and middle covers observed in (A), respectively.

**Figure 5.**
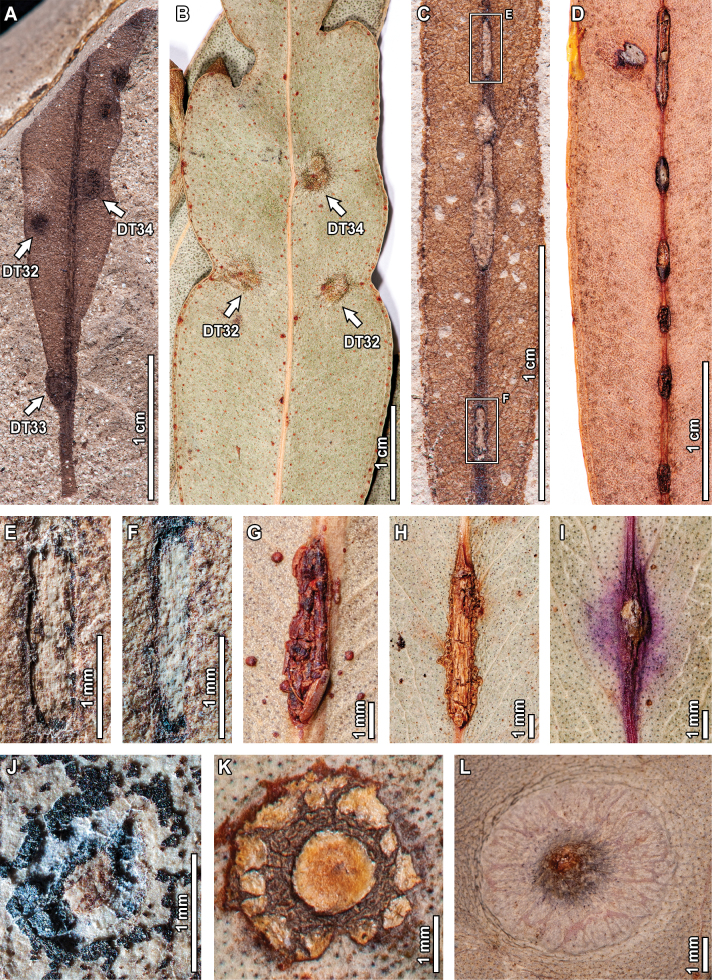
Galling in fossil *Eucalyptus frenguelliana* leaves from Laguna del Hunco (A, C, E, F, J) and corresponding analogs in extant *Eucalyptus* species (B, D, G–I, K, L). A–B. Featureless galls occurring throughout the leaf lamina (DT32), on the midvein (DT33), or along secondary veins (DT34), similarly deforming the leaf’s shape (A. MPEF-Pb 2353; B. *E. punctata* DC. BRI [AQ0838212]); C–I. Series of lenticular galls along the midvein (DT215; C, E, F. MPEF-Pb 2251; D. *E. siderophloia* BRI [AQ0174866]; G. *E. tereticornis* CANB [51504.1]; H. *E. nobilis* CBG [7702033.1]; I. *E. tereticornis* CANB [726772]), (E) and (F) correspond to the lower and upper insets of (C), respectively; J–K. Thick, ellipsoidal galls with inner carbonized cores (DT49; J. MPEF-Pb 2283; K. *E. punctata* CBG [7805142.1]; L. *E. crebra* BRI [AQ0509090]).

**Figure 6.**
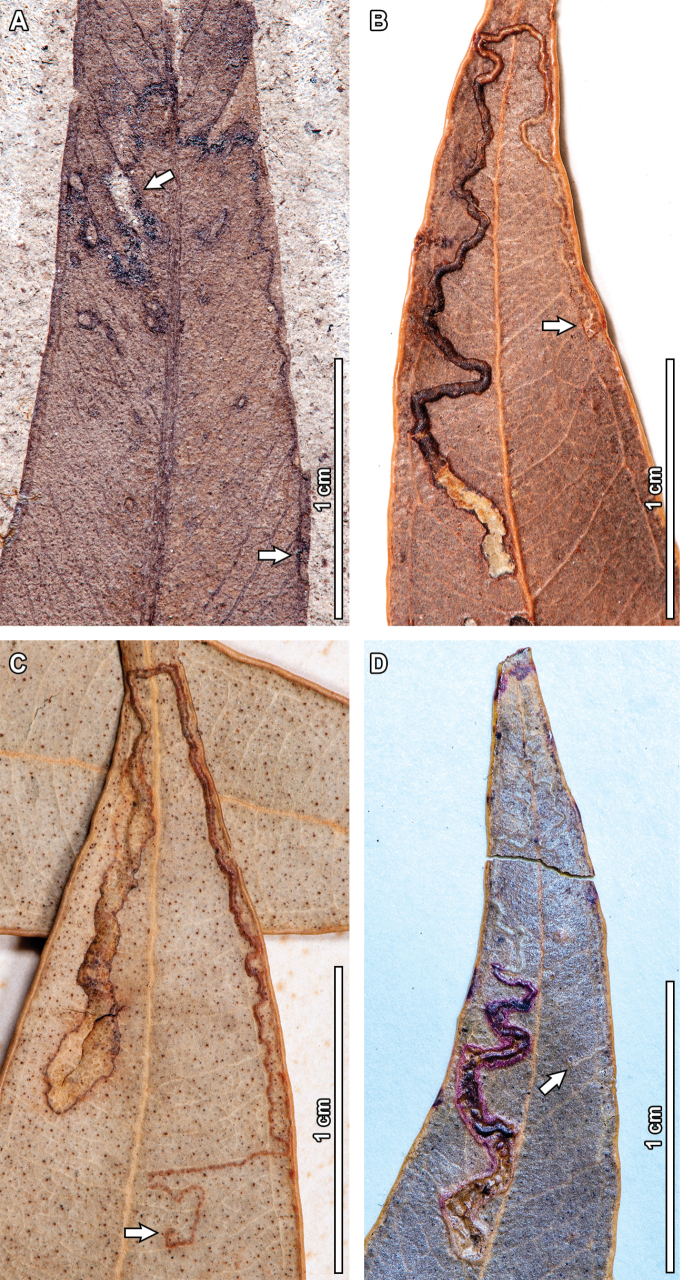
Thread-like mines with a wider phase after crossing the midvein (DT41) in a fossil *Eucalyptus frenguelliana* leaf from Laguna del Hunco (A) and corresponding analogs in extant *Eucalyptus* species (B–D). In (A), lowermost arrow indicates oviposition site, and uppermost arrow the terminal chamber; in (B–D) all arrows indicate oviposition site. A. MPEF-Pb 2358; B. *E. cloeziana* F.Muell. BRI [AQ0097039]; C. *E. punctata* CANB [17112.1]; D. E. *microcorys* A [*C.T.White 1220*, no barcode].

**Figure 7.**
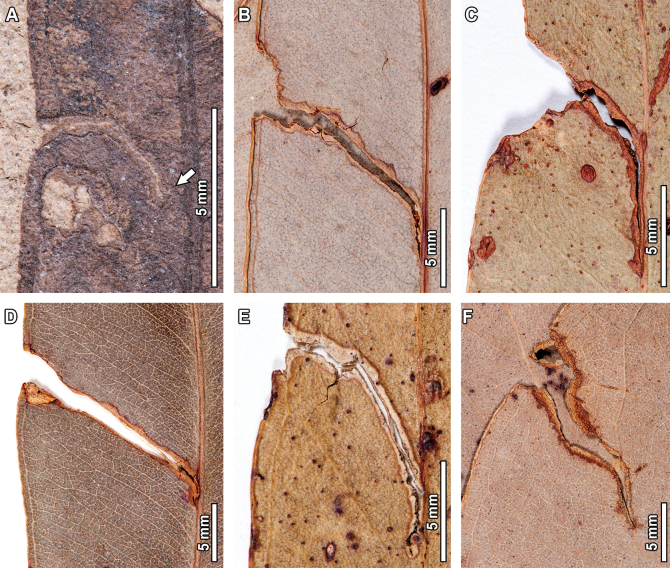
Short curvilinear mines terminating at the leaf margin (DT90) in a fossil *Eucalyptus frenguelliana* leaf from Laguna del Hunco (A) and corresponding analogs in extant *Eucalyptus* species (B–F). In (A), arrow indicates oviposition site. A. MPEF-Pb 2229; B. *E. major* CANB [446889]; C. *E. acmenoides* Schauer BRI [AQ0636111]; D. *E. major* CANB [446889]; E. *E. moluccana* Roxb. CANB [417077]; F *E. platyphylla* F.Muell. BRI [AQ0095472].

**Figure 8.**
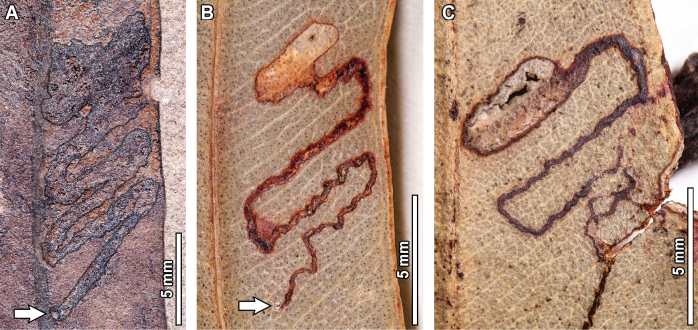
Strongly folded serpentine mines influenced by secondary venation (DT92) in a fossil *Eucalyptus frenguelliana* leaf from Laguna del Hunco (A) and corresponding analogs in extant *Eucalyptus* species (B, C). Arrows indicate oviposition sites, except in the mine depicted in (C), where it is not preserved. A. MPEF-Pb 2347. Note that the midvein, intramarginal and marginal veins, and mine borders were digitally traced to aid in visualization (unedited photo is publicly available online; see Methods); B. *E. major* BRI [AQ0132184]; C. *E. resinifera* CANB [891861.2].

**Figure 9.**
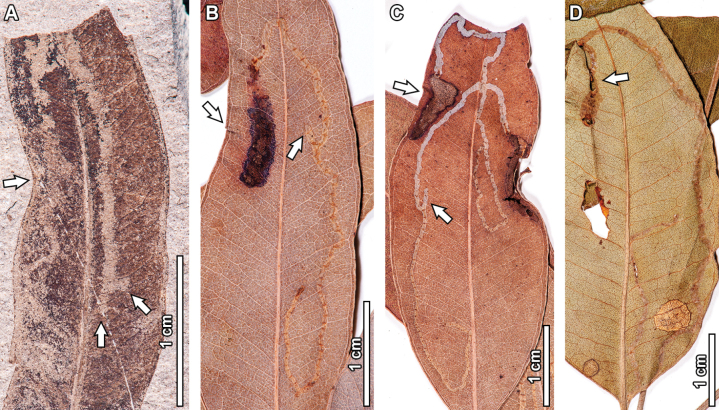
Serpentine mines unaffected by leaf venation and with a generalized lack of frass (DT94) in a fossil *Eucalyptus frenguelliana* leaf from Laguna del Hunco (A) and corresponding analogs in extant *Eucalyptus* species (B–D). Rightmost arrows indicate short bifurcating paths, except in the mine depicted in (D), which lacks one. Leftmost arrows mark the putative pinched leaf margins created by larval behavior (see Discussion). Lowermost arrow in (A) indicates the initiation of the mine. A. MPEF-Pb 13039; B, C. *E. decolor* A.R.Bean & Brooker BRI [AQ0503557]; D. *E. microcorys* BRI [AQ0130317].

**Figure 10.**
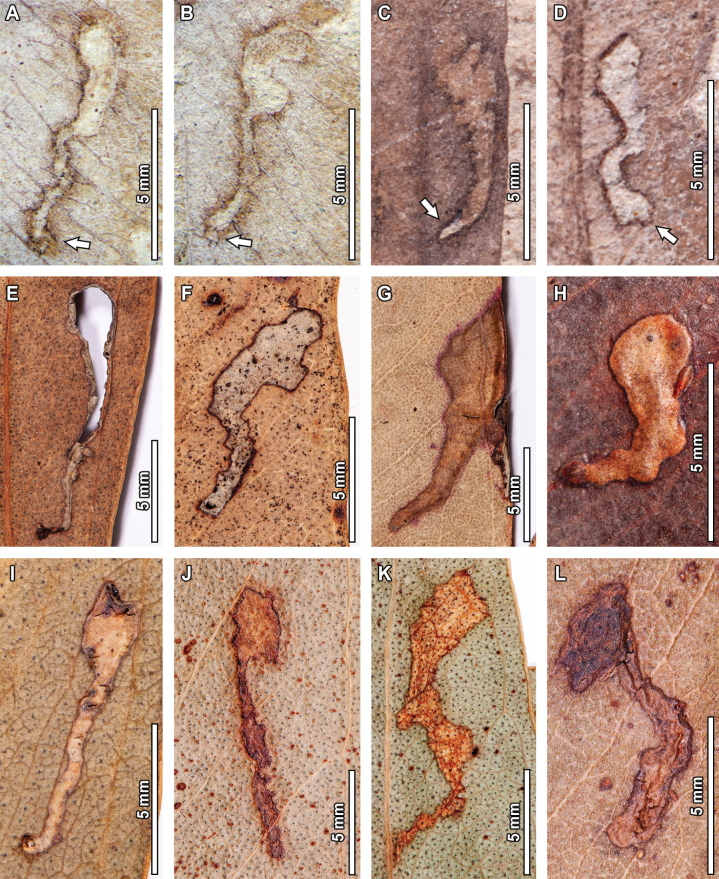
Short curvilinear mines deeply embedded into the leaf tissue (DT139) in fossil *Eucalyptus frenguelliana* leaves from Laguna del Hunco (A–D) and corresponding analogs in extant *Eucalyptus* species (E–L). Lowermost arrows in (A–D) indicate oviposition site, and uppermost arrows in (A–D) the neck of the terminal chamber. A, B. MPEF-Pb 2323; C. MPEF-Pb 2365; D. MPEF-Pb 2336; E. *E. melliodora* A.Cunn. ex Schauer BRI [AQ0130148]; F. *E. crebra* BRI [AQ0097452]; G. *E. fibrosa* BRI [AQ0786999]; H. *E. cloeziana* CANB [123602]; I. *E. melliodora* BRI [AQ0423978]; J. *E. dunnii* Maiden CBG [9504045]; K. *E. nobilis* CANB [725560]; L. *E. acmenoides* CANB [425802].

**Figure 11.**
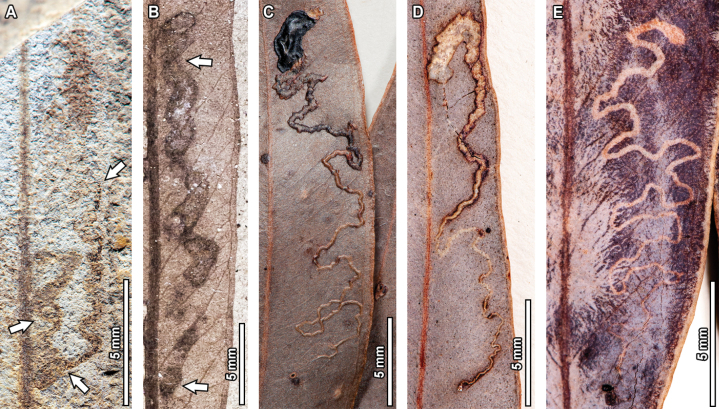
Thin, highly serpentine mines (DT171) in fossil *Eucalyptus frenguelliana* leaves from Laguna del Hunco (A, B) and corresponding analogs in extant *Eucalyptus* species (C–E). In (A), uppermost arrow indicates the initiation of the mine, middle arrow an aborted mine, and lowermost arrow the neck of the terminal chamber. In (B), uppermost arrow indicates the neck of the terminal chamber, and lowermost arrow the oviposition site. A. MPEF-Pb 2247; B. MPEF-Pb 8027; C. *E. cloeziana* CANB [413235]; D. *E. saligna* BRI [AQ0132681]; E. *E. cloeziana* BRI [AQ0097055].

**Figure 12.**
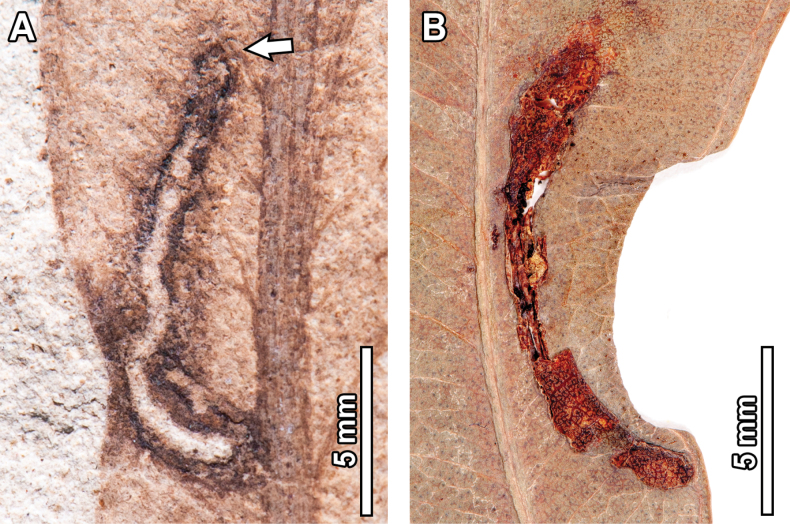
Short curvilinear mines with massive reaction tissue and associated leaf shape deformation (DT185) in a *Eucalyptus frenguelliana* leaf from Laguna del Hunco (A) and corresponding analog in extant *Eucalyptus* species (B). Arrow in (A) indicates the oviposition site. A. MPEF-Pb 2243; B. *E. crebra* BRI [AQ0491261].

**Figure 13.**
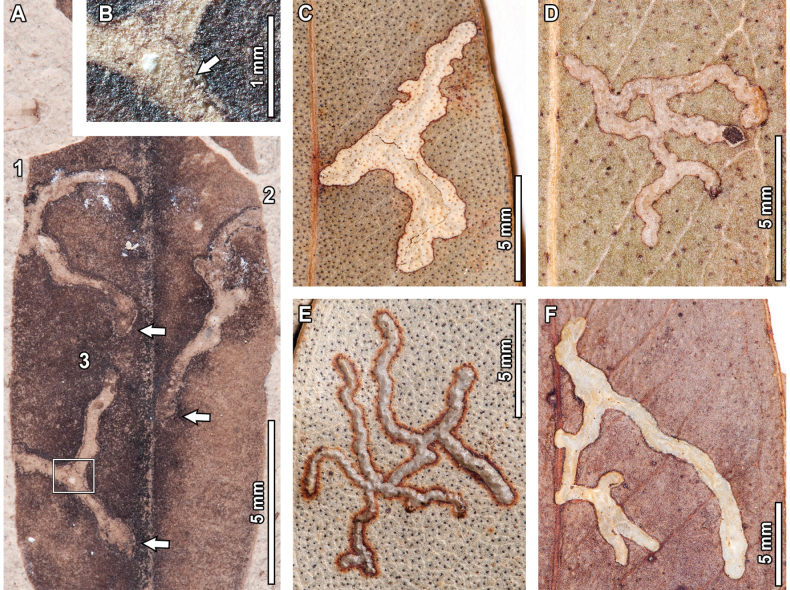
Mines with smooth borders and frequent bifurcating paths (DT207) in a fossil *Eucalyptus frenguelliana* leaf from Laguna del Hunco (A, B) and corresponding analogs in extant *Eucalyptus* species (C–F). Notice the thin, fluidized frass trail on one side of the mine highlighted by the arrow in (B), a close-up of the inset of the mine marked as “3” in (A). Arrows in (A) indicate the initiation of the mines (see Results). A, B. MPEF-Pb 13041; C. *E. punctata* BRI [AQ0132345]; D. *E. tindaliae* Blakely BRI [AQ0727591]; E. *E. punctata* CANB [456662]; F. *E. oreades* R.T.Baker BRI [AQ0131070].

**Figure 14.**
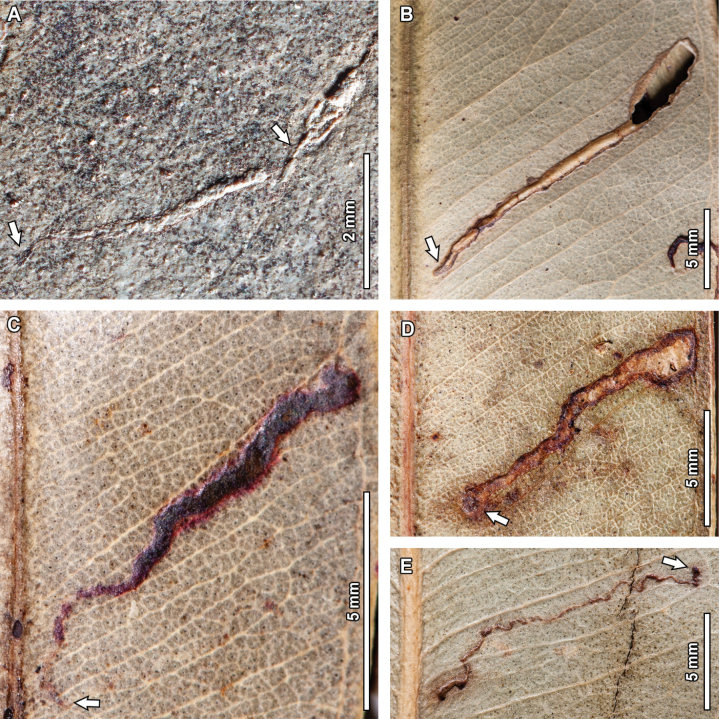
Short mines circumscribed by two secondary veins (DT210) in a fossil *Eucalyptus frenguelliana* leaf from Laguna del Hunco (A) and corresponding analogs in extant *Eucalyptus* species (B–E). In (A), uppermost arrow indicates the neck of the terminal chamber, and lowermost arrow the oviposition site. In (B–E), all arrows indicate oviposition site. A. MPEF-Pb 2321; B. *E. robusta* CANB [455708]; C. *E. resinifera* BRI [AQ0657633]; D. *E. grandis* CANB [135660.1]; E. *E. robusta* CANB [455769].

**Figure 15.**
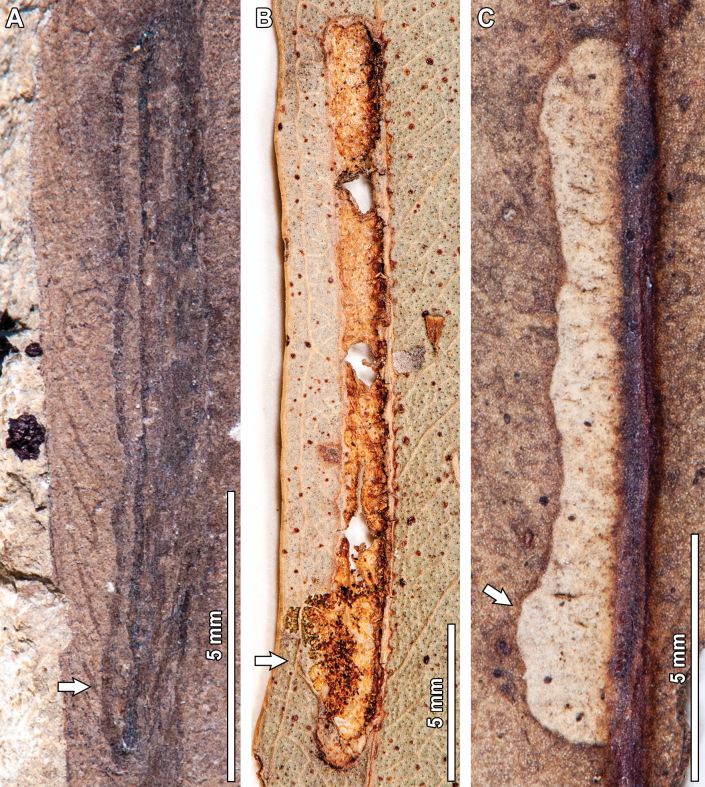
Mines with constant width occurring alongside the midvein (newly described DT422) in a fossil *Eucalyptus frenguelliana* leaf from Laguna del Hunco (A) and corresponding analogs in extant *Eucalyptus* species (B, C). Arrows indicate terminal chambers. The terminal chamber of the mine depicted in (B) was probably breached due to environmental factors. A. MPEF-Pb 13038; B. *E. tereticornis* CBG [35770.1]; C. *E. moluccana* BRI [AQ0130252].

**Figure 16.**
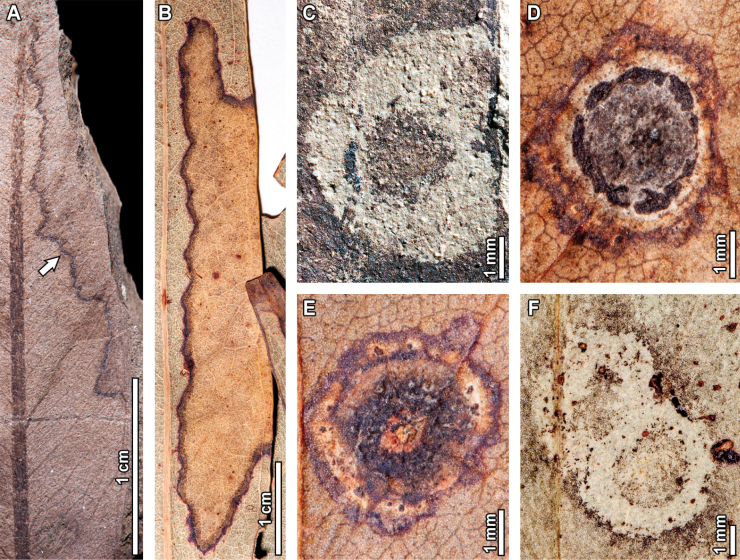
Pathogenic damage in fossil *Eucalyptus frenguelliana* leaves from Laguna del Hunco (A, C) and corresponding analogs in extant *Eucalyptus* species (B, D–F). A, B. Necrotic tissue occurring along the leaf margin (DT114) with thick reaction fronts (A. MPEF-Pb 13036; B. *E. michaeliana* CANB [154381.1]); C–F. Circular fungal blotches with prominent, carbonized cores (DT261) occurring singly (C–E) or in couplets (F) (C. MPEF-Pb 13037; D, E. *E. cloeziana* CANB [413275]; F. *E. moluccana* BRI [AQ0820055]).

**Figure 17.**
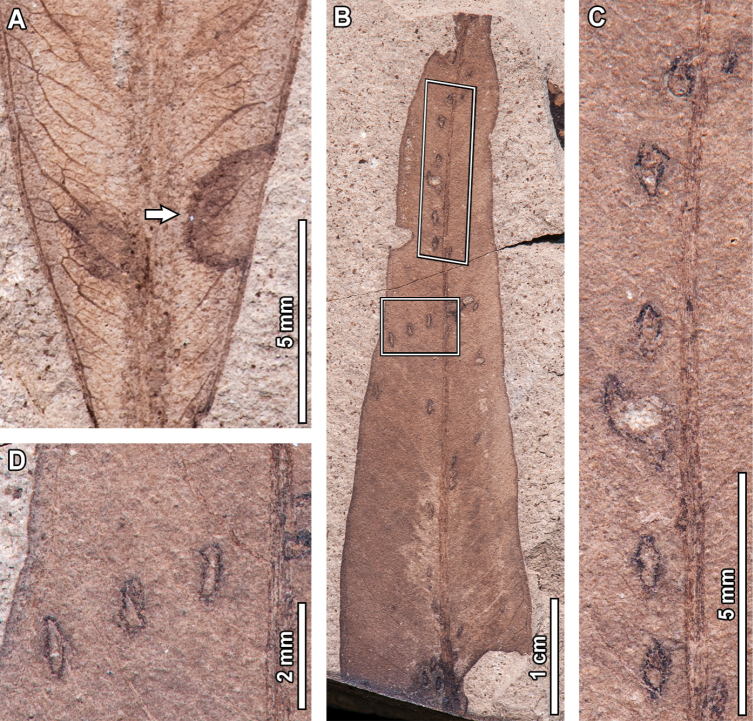
Pathogenic damage and oviposition scars in *Eucalyptus frenguelliana* leaves from Laguna del Hunco without extant counterparts. A. Ovoidal blotch of necrotic tissue originating near the midvein (see arrow; DT58; MPEF-Pb 2305); B. Rows of elliptical scars occurring throughout the leaf lamina (DT54; lowermost inset), and lenticular scars aligned end-to-end as a single row paralleling the midvein (DT310; uppermost inset; MPEF-Pb 2216); C. Detail of DT310 oviposition scars in (A), photographed and figured independently by Sarzetti et al. (2009: fig. 2.3, 4; MPEF-IC 1376) and assigned to the ichnotaxon *Paleoovoidus rectus*; D. Detail of DT54 oviposition scars in (A), photographed and figured independently by Sarzetti et al. (2009: fig. 2.4; MPEF- C 1392) and assigned to the ichnotaxon *P. arcuatum* of Sarzetti et al. (2009: fig. 2.4; MPEF-IC 1392).

The 33 damage types (DTs) identified in the full collection of 284 fossil *Eucalyptus frenguelliana* leaves from LH include twelve types of external feeding (Figs 1–3), one of piercing-and-sucking (Fig. 4), five of galling (Fig. 5), ten mining (Figs 6–15), three of pathogenic traces (Figs 16, 17A), and two of oviposition scars (Fig. 17B–D). In the external feeding category, three of the associations refer to hole feeding (Fig. 1), five to margin feeding (Fig. 2), two to surface feeding (Fig. 3A–E), and two to skeletonization (Fig. 3F–J). The majority of fossil leaves (ca. 66%; 189 specimens) have at least one DT, and up to seven DTs were recorded in a single leaf (Suppl. material 1: dataset S2).

Each of the 28 insect herbivory DTs identified in the fossil material has a corresponding analog in the extant *Eucalyptus* species surveyed (Figs 1–15; Giraldo et al. 2025). Below, I provide detailed morphological descriptions for all the DTs identified in the fossils, followed by photographic documentation and comparisons with leaf damage observed on extant *Eucalyptus* herbarium specimens that resemble very closely the leaf damage of the *E. frenguelliana* fossils. Fossil leaf mine descriptions and comparisons with extant analogs are highly detailed because the insects that produce them are typically host-specific and leave distinct mining traces, and the presence of morphologically similar mines on taxonomically related plant hosts provides the most convincing evidence for host-tracking through geologic time (Donovan et al. 2020, 2023; Giraldo et al. 2025). Because over 10,000 herbarium sheets were reviewed (see Methods), it was not feasible to score all sheets for all DTs, and many of the herbarium specimens have additional DTs. I also mention the most prominent *Eucalyptus* insect folivores for each herbivory category based on an extensive review of the literature.

### ﻿External feeding

Fossil *Eucalyptus* specimens include circular-to-ellipsoidal holes measuring 0.3–0.9 mm in diameter (DT1, Fig. 1A); polylobate holes 2.1–4.8 mm in length and 1.1–1.4 mm in width (DT3, Fig. 1A); and elongate holes with parallel sides, 0.6–2.5 mm in length and 0.4–1.2 mm in width (DT8, Fig. 1E). Margin feeding includes excisions removing the leaf apex (DT13, Fig. 2A), 9.2–10.1 mm deep; semicircular shallow excisions along the leaf margin (DT12, Fig. 2D) that measure 0.6–10.1 mm in width and are 0.6–6.4 mm deep; and consecutive, nearly perfect semicircular excisions (DT81, Fig. 2G) that measure 3.43–8.4 mm in width and are 2.1–6.3 mm deep. Margin feeding traces reaching the midvein (DT14, Fig. 2J) measure 1.4–48.9 mm in width and are 0.8–3.8 mm deep; and those that expand towards the midvein (DT15, Fig. 2M) measure 5.7–9.4 mm in width and are 3.9–6.3 deep. Surface feeding encompasses polylobate (DT30, Fig. 3A) and circular (DT31, Fig. 3C) abrasions, measuring 3.3–5.4 mm in length and 1.7–2.2 mm in width in the former, and 0.6–0.7 mm in diameter in the latter. Skeletonization includes irregularly shaped patches with removed interveinal tissue and a faint reaction rim (DT16, Fig. 3F), measuring 3.3–21.8 mm in length and 2.1–2.8 mm in width; and those with thick reaction rims (DT17, Fig. 3H), measuring 1.3–10.2 mm in length and 0.5–6.3 mm in width. Reaction rims surrounding external feeding traces are 0.1–0.2 mm thick.

The small circular (DT1) and polylobate holes (DT3) found in the fossils (Fig. 1A) are commonly found in extant *Eucalyptus* (Fig. 1B–D), with similar locations along the leaf lamina. Elongate holes (DT8; Fig. 1E) are also abundant in extant specimens, although they tend to be associated with leaf shape deformation (Fig. 1F–H), a feature not observed in the fossils. Margin excisions are very commonly found in extant *Eucalyptus*, with modern analogs for leaf apex removal (DT13; Fig. 2B, C), shallow excisions along the leaf margin (DT12; Fig. 2E, F), consecutive and nearly perfect semicircular excisions (DT81; Fig. 2H, I), excisions reaching the midvein (DT14; Fig. 2K, L)—with some extreme examples where almost all leaf tissue is removed (Fig. 2L)—and deeply trenched excisions with expansions towards the midvein (DT15; Fig. 2N). Surface abrasions and skeletonization traces are much less frequent than hole and margin feeding in both fossil and extant specimens, but analog damage was still observed for polylobate (DT30; Fig. 3B) and circular (DT31; Fig. 3D, E) surface feeding traces, as well as for skeletonized patches with a faint reaction rim (DT16; Fig. 3G) and those with a thick reaction tissue (DT17; Fig. 3I, J).

Today, the leaves of *Eucalyptus* are chewed by a wide range of insect species pertaining to several orders such as Hymenoptera, Orthoptera, Phasmatodea, Thysanoptera, and especially Coleoptera and Lepidoptera (see Dataset S4 of Giraldo et al. 2025). Major pests include sawflies in the genus *Perga* (Pergidae), stick insects in the genus *Didymuria* (Phasmatidae), and several species of *Anoplognathus* (Scarabaeidae) (Moore 1972; Neumann et al. 1977; Carter et al. 1981; Edwards et al. 1993; Carnegie 2008; Jones et al. 2015). Less harmful herbivores include several species of *Paropsis*, *Paropsisterna*, and *Trachymela* in the beetle family Chrysomelidae (Carter et al. 1981; Tanton and Epila 1984; Tribe and Cillie 1997), as well as hundreds of lepidopteran species, many of which remain undescribed, in families such as Erebidae, Gelechiidae, Geometridae, and Limacodidae, among others (Moore 1972; Common 1990).

### ﻿Piercing-and-sucking

Piercing-and-sucking traces in the fossil material include circular scale insect covers occurring in clusters (DT77, Fig. 4A–C). The covers measure 0.6–0.9 mm in diameter and have up to 5 concentric growth rings (Fig. 4C). Similar covers were observed in multiple leaves of one *E. notabilis* Maiden herbarium specimen (Fig. 4D, E). Although the individual covers are 2–2.5 times bigger in the extant specimens when compared to the fossil counterparts, there is a close similarity in terms of shape, waxy texture, and presence of concentric growth rings.

An extremely diverse array of piercing-and-sucking hemipterans attack extant *Eucalyptus* species, with most of the reported associations pertaining to the families Diaspididae, Eriococcidae, and Psyllidae, some of which have become pests in *Eucalyptus* plantations (Carnegie 2008; Jones et al. 2015). Widely associated genera include *Chrysomphalus* and *Neoleonardia* in Diaspididae (Brimblecombe 1957, 1958, 1962; Normark et al. 2019); *Acanthococcus*, *Eriococcus*, and *Phacelococcus* in Eriococcidae (Hoy 1963; Zondag 1977; Curry 1981; Wylie and Bevege 1981; Gullan and Vranjic 1991; Phillips 1993; Whitham et al. 1994); and *Anoeconeossa*, *Blastopsylla*, *Cardiaspina*, and *Glycaspis* in Psyllidae (Taylor 1962, 1985, 1987; Moore 1970). In a classical paper, Moore (1961) suggested that the high richness of psyllids, particularly those in the genus *Glycaspis*, is probably the result of co-diversification with their *Eucalyptus* hosts. Other hemipterans attacking *Eucalyptus* include leafhoppers (Cicadellidae), cicadas (Cicadidae), and leaf-footed bugs (Coreidae), among others (Moore 1972; Carnegie 2008; Jones et al. 2015). Over 600 hemipteran-to-*Eucalyptus* associations were reported in Dataset S4 of Giraldo et al. (2025), dozens of which are also compiled and routinely updated in https://scalenet.info/ (García Morales et al. 2016).

### ﻿Galling

Fossil *E. frenguelliana* leaves have several galling DTs, including circular-to-ellipsoidal galls without distinctive features (Fig. 5A) positioned along the lamina (DT32; 0.7–1.5 mm long by 0.2–0.8 mm wide), as well as primary (DT33; 1.4–3.1 mm long by 0.6–1.3 mm wide) and secondary veins (DT34; 0.6–2.1 mm in diameter). Series of up to four lenticular galls occurring along major veins (DT215, Fig. 5C, E, F) are 1.4–4.9 mm in length and 0.4–0.8 mm in width, separated by gaps of up to 7 mm. Ellipsoidal galls with a large inner carbonized core and a featureless encircling area (DT49, Fig. 5J) measure 3.3–3.5 mm in length and 1.4–1.8 mm in width, with a prominent 0.2 mm thick reaction rim and the core measuring 0.5–1.7 mm in diameter.

Featureless galls on the leaf lamina (DT32) are common in extant *Eucalyptus*, much more so than those occurring along primary (DT33) or secondary (DT34) veins. Whether fossil (Fig. 5A) or extant (Fig. 5B), these featureless galls frequently deform the surrounding leaf tissue, seen as indentations along the leaf margin, and deflect leaf venation. Series of lenticular galls occurring on the midvein (DT215) are also observed in extant specimens (Fig. 5D, G–I), sharing several morphological features with the fossil exemplars such as the spacing along the midvein (Fig. 5C, D), as well as having individual galls with length-width ratios of 3:1 and an irregular, jagged reaction rim (Fig. 5E–I). The DT215 galls are, however, slightly bigger in the extant exemplars when compared to those of the fossils, something that is also seen for ellipsoidal galls with large, inner carbonized cores (DT49; Fig. 5J–L), wherein their extant counterparts (Fig. 5K, L) are larger, but otherwise share morphological similarities such as an outer rim that encloses a ring of wrinkled tissue, and a circular core with radiating tissue masses.

Gall-inducing insects attacking extant *Eucalyptus* species have received far less attention when compared to piercing-and-sucking insects, yet representatives are found across Coleoptera, Diptera, and especially Hemiptera and Hymenoptera. Dozens of scale insect (Eriococcidae) species also induce galls in *Eucalyptus*, frequently producing dimorphic galls that are well-documented in the literature (e.g., Cook 2003; Hardy and Gullan 2010; Hardy et al. 2019). On the other hand, despite being native to Australia, several gall-inducing wasps have been described from *Eucalyptus* plantations in Europe (Borowiec et al. 2019), the Middle East (Mendel et al. 2004; Protasov et al. 2007; Dittrich-Schröder et al. 2020), and South America (Molina-Mercader et al. 2019), and at least 320 species still await description (Dittrich-Schröder et al. 2020).

### ﻿Mining

#### ﻿DT41

In one of the fossil leaves, there is one instance of a serpentine, frass-filled mine with a gradual increase in width and an elliptical terminal chamber, assigned to DT41 (Fig. 6A). Preserved length is 39.5 mm and width ranges between 0.1–0.7 mm. The mine has a distinctive, circular oviposition site—0.5 mm diameter—nearby the leaf margin (see lowermost arrow in Fig. 6A). The mine follows a curvilinear path towards the leaf apex for 18.1 mm at a constant width of 0.1 mm and is slightly deflected by secondary veins. The mine then turns 90° counterclockwise and follows a 3.7 mm long path towards the midvein in a curvilinear manner, with a slight width increase to 0.2 mm. The mine then follows the midvein towards the leaf apex for another 2.3 mm before turning 90° counterclockwise, after which it follows an additional 7.2 mm long curvilinear path directed towards the left margin of the leaf, at a ~135° angle with respect to the midvein. After reaching the submarginal vein, the mine turns 45° counterclockwise and follows a 3.9 mm long curvilinear path towards the midvein, reaching a width of 0.6 mm. After a tight U-turn, the mine continues for a short, 1.5 mm linear path before reaching the neck of the terminal chamber. The breached terminal chamber (see uppermost arrow in Fig. 6A) is elliptical in shape, 2.3 mm in length and 0.9 mm in width, and is completely circumscribed by two secondary veins. The mine is filled with frass throughout its course, and individual pellets are spheroidal-to-ellipsoidal in shape, 0.1 mm in length and 0.05 mm in width.

Three extant analogs were found for this mine (Fig. 6B–D). Fossil and extant mines share having an initial phase characterized by a very thin, thread-like path that mostly occurs between the intramarginal vein and the leaf margin; and a subsequent, wider phase that takes place after the mine crosses the midvein near the leaf apex. The mines also share a circular oviposition site, 0.5 mm in diameter, and a broadly elliptical terminal chamber (except in the mine depicted in Fig. 6B, where the terminal chamber is elongated). The extant analogs in Fig. 6C, D differ from the fossil mine in not having the oviposition site between the intramarginal vein and the leaf margin (see arrows in Fig. 6), and, in the case of the mine in Fig. 6C, in having a blotch-like phase (instead of a serpentine phase) after crossing the midvein.

#### ﻿DT90

One fossil *E. frenguelliana* leaf has a linear mine terminating at the leaf margin, characterized by irregular, ragged margins (DT90, Fig. 7A). Preserved length is 5.2 mm and width ranges between 0.5–0.9 mm. The mine has a circular-to-subrounded oviposition site beside the midvein (see arrow in Fig. 7A), measuring 0.9 mm in diameter. The first 2.1 mm of the mine have a constant width of 0.6 mm and follow a linear path at a ~35° angle with respect to the midvein. The mine then turns ~55° counterclockwise, becoming perpendicular with respect to the midvein, and follows a 2.2 mm path towards the leaf margin, where it ultimately terminates. During this last portion of the mine, there are sudden width expansions (up to 0.9 mm) and constrictions (down to 0.5 mm). Throughout the course of the mine, there is a whitish, central, apparently fluidized strand of frass, 0.1 mm in width, as well as a distinct 0.1 mm thick reaction rim in the periphery of the mine.

Five extant analogs were found for DT90 (Fig. 7B–F). Shared features between the fossil and extant mines include having a very short curvilinear path that starts beside the midvein (but see the exception below), a somewhat irregular border, and a sudden width expansion before terminating in the leaf margin. In the extant mines, the damaged areas are flanked by flaps of epidermal tissue, which I interpret as breaching due to environmental factors such as in vivo abrasion. Minor differences between the fossil and extant mines refer to having a more linear path (for the mine seen in Fig. 7D) and in starting beside a secondary vein instead of the midvein (for the mine depicted in Fig. 7F).

#### ﻿DT92

A highly folded, serpentine mine, circumscribed between the midvein and leaf margin and influenced by secondary venation was observed in the leaf fossils (Fig. 8A). The mine, assigned to DT92 has a preserved length of 38.9 mm and its width ranges between 0.4–0.7 mm. The mine has a distinctive circular oviposition site adjacent to the midvein (see arrow in Fig. 8A), measuring 0.7 mm in diameter. The initial 1.5 mm of the mine are 0.4 mm in width, after which the mine abruptly widens to 0.7 mm; this width is maintained for the rest of the mine’s course. The mine then follows a secondary vein for 6.1 mm until it reaches the leaf margin, turns 180°, and tracks the superjacent secondary vein for 6.5 mm until reaching the midvein. After contacting the midvein, the mine turns around and goes through another cycle of following a secondary vein for 7 mm until reaching the leaf margin, turning 180°, and tracking the superjacent secondary vein for 4.9 mm until reaching the midvein. After this second contact with the midvein, the mine tracks the next corresponding secondary vein for 5.8 mm before reaching the roughly elliptical terminal chamber, 6.3 mm in length and 3 mm in width. The full width of the mine is filled with spheroidal frass pellets (0.1 mm in diameter) that give the mine its carbonized appearance.

Two extant analogs were observed for this mine (Fig. 8B–C). Fossil and extant mines share several similarities, such as being bounded by the midvein and intramarginal vein, having a path that is dictated by the presence of secondary veins, a terminal chamber that is elliptical in shape, and frass that occupies the full width of the mine (thus giving the mine a dark, carbonized appearance). The extant analogs differ from the fossil mine in having oviposition sites in other places along the leaf lamina, or, for the mine depicted in Fig. 8B, in not contacting the midvein.

#### ﻿DT94

Another fossil serpentine mine, characterized by irregular borders, a bifurcating path, and crossing of the midvein is assigned to DT94 (Fig. 9A). Total preserved length is 46 mm, and width ranges between 0.6–2.2 mm. The mine has no discernible oviposition site, perhaps due to preservational artifacts, but seems to initiate close to the midvein (see lowermost arrow in Fig. 9A). The initial 17.5 mm of the mine are directed towards the leaf apex, with a constant width of 0.9 mm; although there is a short, 1.3 mm bifurcation nearby the start of the mine (see middle arrow in Fig. 9A). The mine then crosses the midvein and turns 180°, reorienting towards the leaf base. This U-turn is 3.4 mm in length and up to 2.2 mm in width, although the apparent width increase could be due to a patchy preservation. After the U-turn, the mine follows a linear path for 8.4 mm, which becomes sinusoidal for the next 12.6 mm, with a slight decrease in width to 0.6 mm. The sinusoidal phase ends beside the midvein, where the width increases to 0.9 mm before reaching the terminal chamber. In turn, the terminal chamber is elliptical in shape, 4.1 mm in length and 2.3 mm in width, and occupies the entire width of the leaf between the midvein and the leaf margin. No frass is preserved.

Three extant analogs were observed for DT94. Fossil and extant mines share several features, such as the short bifurcating paths (see arrows in Fig. 9) in the initial phases of the mine (except in the mine in Fig. 9D), crossing of the midvein without deflection of the mine path, elliptical terminal chambers (but see exception below), and a generalized lack of frass. Extant examples differ from the fossil in having more clearly defined mine borders, although the difference could be due to suboptimal preservation in the fossil mine; in terminating shortly after crossing the midvein, and, for one of the mines depicted in Fig. 9C, in having a terminal chamber that is more triangular than elliptical in shape.

#### ﻿DT139

Multiple *E. frenguelliana* leaves have short, curvilinear mines deeply embedded in the leaf tissue and generally unaffected by leaf venation (DT139, Fig. 10A–D). The first instance (Fig. 10A) has a preserved length of 7.4 mm, and its width ranges between 0.3–0.8 mm. The mine has a distinctive polygonal oviposition site (see lowermost arrow in Fig. 10A), 0.6 mm in length and 0.2 mm in width. The mine is directed towards the leaf apex, and the initial 3.8 mm have a slight width increase from 0.3 mm to 0.4 mm. The mine then becomes blotch-like for 1.8 mm, and its width increases to 0.8 mm before reaching the terminal chamber (see uppermost arrow in Fig. 10A). The terminal chamber is elliptical in shape, 1.2 mm in length and 1 mm in width. A distinct, 0.1 mm thick reaction rim is present throughout the mine, as well as a 0.2 mm wide diffuse necrotic tissue. Sporadic, ellipsoidal frass pellets occur in the blotch-like phase, 0.1 mm in length and 0.05 mm in width.

The second instance of DT139 (Fig. 10B) has a preserved length of 6.1 mm, and its width ranges between 0.4–0.5 mm. The mine has a distinctive polygonal oviposition site (see lowermost arrow in Fig. 10B), 0.1 mm in length and 0.4 mm in width. The mine then follows a linear path for 3.5 mm, with minimal width increases from 0.4 to 0.5 mm, before reaching the terminal chamber. The terminal chamber (see uppermost arrow in Fig. 10B) is dumbbell-shaped, suggesting that it was most likely aborted before being fully formed. If completed, the terminal chamber would have been elliptical in shape, 2.5 mm in length and 1.6 mm in width. A distinct 0.1 mm thick reaction rim is present throughout the mine, as well as a 0.2 mm wide diffuse necrotic tissue. Frass is only present in the terminal chamber, where sporadic spheroidal-to-ellipsoidal pellets (0.1 mm in length and 0.05 mm in width) are present.

The third instance of DT139 (Fig. 10C) has a preserved length of 6.9 mm and its width ranges between 0.2–1 mm. In contrast to the last two instances, this mine lacks a distinct oviposition site, but its course starts besides the midvein and is initially breached (see arrow in Fig. 10C). The mine follows a linear path towards the leaf margin for 1.5 mm, circumscribed by two secondary veins and increasing in width from 0.2 mm to 0.6 mm. The mine then turns towards the leaf apex and follows a linear path for 2.8 mm before reaching the neck of the terminal chamber. At the end of this portion, the mine is 1 mm in width. The terminal chamber is roughly rectangular in shape, 2.6 mm in length and 2 mm in width, with somewhat irregular borders due to the presence of secondary veins. There is a 2 mm thick reaction tissue in the first half of the mine, which becomes increasingly diffuse in the second half. Frass is absent.

The fourth and final instance of DT139 (Fig. 10D) has a preserved length of 4.8 mm and its width ranges between 0.5–1 mm. The mine has a distinctive, rectangular oviposition site (see lowermost arrow in Fig. 10D), 0.4 mm in length and 0.2 mm in width. The mine then follows a linear path towards the leaf apex for 1.6 mm, with a width decrease from 1 mm in the first half to 0.5 mm in the second half. After this, the mine turns ca. 45° clockwise and moves towards the leaf margin for 1 mm—at a constant width of 0.6 mm—before reaching the neck of the terminal chamber (see uppermost arrow in Fig. 10D). In turn, the terminal chamber is polylobate in shape, 2 mm in length and 1.4 mm in width. A distinct reaction rim, 0.2 mm wide is present throughout the left margin of the mine. Frass is absent.

Eight extant analogs were observed for the DT139 mines (Fig. 10E–L). Fossil and extant mines share being very short in total length (< 1 cm), having a curvilinear path that is generally unaffected by leaf venation, and being deeply embedded into the leaf tissue. There are, however, some minor differences between the extant examples when compared to the fossil mines, such as being present in the upper layers of leaf tissue (Fig. 10F, G, K) or being slightly deflected by secondary veins (Fig. 10K), although this is also seen for the fossil mine in Fig. 10C.

#### ﻿DT171

Two highly serpentine mines with rounded, smooth borders (DT171) were observed in the fossil material (Fig. 11A, B). The first instance of DT171 (Fig. 11A) has a preserved length of 11 mm (measured from where the path can be reconstructed; see uppermost arrow in Fig. 11A), and its width ranges between 0.1–0.4 mm. The mine lacks a distinctive oviposition site, possibly due to preservational artifacts, but it seems to start somewhere near the leaf margin in the apical portion of the leaf. The mine follows a highly coiled, serpentine path for 5.8 mm, with several tight turns in the middle portion and a width that ranges between 0.1–0.3 mm. The mine then follows a sinusoidal-meandering path for 3.8 mm, with a slight width increase to 0.4 mm before reaching the neck of the terminal chamber. In turn, the terminal chamber (see lowermost arrow in Fig. 11A) is elliptical in shape, 1.4 mm in length and 0.6 mm in width. Frass occupies the entire width of the mine, with individual pellets being circular in shape and < 0.1 mm in diameter. The structure occurring beside the midvein (see middle arrow in Fig. 11A) is interpreted as an aborted mine that is not described or consider further, given its generalized lack of structural features and inability to track its course.

The second instance of DT171 (Fig. 11B) has a preserved length of 30 mm, and its width ranges between 0.9–1.3 mm. The mine has a distinctive elliptical oviposition site (see lowermost arrow in Fig. 11B) beside the midvein, 1.8 mm in length and 0.9 mm in width. After following a meandering path for 25.3 mm, with a minor width increase from 0.9 mm to 1.4 mm, the mine reaches the neck of the terminal chamber (see uppermost arrow in Fig. 11B). The terminal chamber is roughly elliptical in shape, 2.9 mm in length and 1.5 mm in width. Frass occupies the entire width of the mine, and individual frass pellets are elliptical in shape, 1.2 mm in length by 1 mm in width. A distinctive, 0.1 mm thick reaction tissue is present throughout the course of the mine.

Three extant analogs were observed for DT171 (Fig. 11C–E). Commonalities between fossil and extant mines include being highly serpentine, occurring on one side of the leaf midvein, having a path that is unaffected by leaf venation, an elliptical terminal chamber, and smooth rounded borders. Minor differences seen for the extant examples when compared to the fossil mines include lack of frass (for the mine depicted in Fig. 11E), and having elongated terminal chambers (for the mine in Fig. 11D).

#### ﻿DT185

A curvilinear mine with rounded borders and massive reaction tissue that deforms leaf shape and venation (DT185; Fig. 12A) was observed in the fossil *Eucalyptus* leaves. Preserved length is 14.7 mm, and width ranges between 1–1.2 mm. The mine lacks a distinguishable oviposition site but starts near the midvein (see arrow in Fig. 12A). The first 10 mm of the mine follow a linear path towards the leaf margin, at an angle of ~160° with respect to the midvein. This first portion has a constant width of 1 mm, and the reaction tissue is 0.2 mm wide. The mine then turns ~90° counterclockwise and follows a linear path towards the midvein for 4.7 mm. Leaf shape and venation are clearly deformed by this turn. At the final portion of this segment, the mine is 1.2 mm wide, and the reaction tissue becomes massive, up to 1 mm in thickness. There is no terminal chamber or frass present in the mine.

One extant analog was observed for DT185 (Fig. 12B). The fossil and extant mines share having short (<2 cm) curvilinear paths that are deeply embedded in the leaf tissue and producing a massive reaction rim that deforms leaf shape. However, the extant mine terminates facing the leaf margin, whereas the fossil mine finishes in contact with the midvein.

#### ﻿DT207

One fossil *E. frenguelliana* specimen has three instances of DT207 (Fig. 13A), which are mines characterized by smooth borders, a thin frass trail abutted to one of the mine’s margins (Fig. 13B), and frequent bifurcations and path reversals. The mine in the upper left of the leaf (see “1” in Fig. 13A) has a preserved length of 10.3 mm and its width ranges between 0.3–0.5 mm. Despite lacking a distinctive oviposition site, the mine seems to start beside the midvein (see uppermost arrow in Fig. 13A), following a curvilinear path for 5.2 mm towards the leaf margin. The mine is 0.3 mm wide in this portion, but small, 0.2 mm cusps are commonly seen protruding from the borders of the mine. The path of the mine is then reoriented towards the midvein for another 5.1 mm. There is no presence of a terminal chamber.

The second DT207 mine, located in the right side of the leaf (see “2” in Fig. 13A), has a preserved length of 8.3 mm, and its width varies between 0.5–0.8 mm. Although the mine lacks an oviposition site, the thinnest portion of the mine (see middle arrow in Fig. 13A) is interpreted as the starting point. The mine follows a 4.4 mm long curvilinear path towards the leaf margin at a ~25° angle with respect to the midvein. The mine is 0.5 mm wide in this first portion, although sporadic, 0.2 mm cusps extend the mine’s width to 0.7 mm. The mine then turns towards the leaf apex for 1.1 mm, before following a 2.8 mm curvilinear path directed towards the leaf margin, where it ultimately terminates. During this second portion of the mine, width ranges between 0.5–0.7 mm, although there is a sudden width expansion to 0.8 mm right at the leaf margin. There is no terminal chamber.

Finally, the third instance of DT207 seen in the lower-left portion of the leaf (see “3” in Fig. 13A) has a preserved length of 8.3 mm, and its width ranges between 0.4–0.9 mm. The mine has a putative elliptical oviposition site (see lowermost arrow in Fig. 13A), 1.4 mm in length and 0.8 mm in width. After following a linear path for 1.6 mm, at a ~30° angle with respect to the midvein, the mine bifurcates. One path follows a linear trail for 1.6 mm until reaching the leaf margin, where it ultimately ends, with a constant width of 0.5 mm except at the contact with the margin, where it expands to 0.9 mm width. The other path follows a 3.7 mm long trail at a ~140° angle with respect to the midvein, with a constant width of 0.5 mm. As with the previous two mines, there is no presence of a terminal chamber. All three mines have a 0.05 mm wide frass trail abutted to one of the mine’s borders (see Fig. 13B), as well as a 0.05 mm thick reaction tissue

Four extant analogs were observed for these bifurcating mines (Fig. 13C–F). Fossil and extant mines share having frequently bifurcating paths, being positioned besides the midvein, and having smooth borders with frequent cusps. The extant examples differ from the fossils in not having a clearly visible frass trail that occurs on one side of the mine, and, in the examples depicted in Fig. 13D–F, there are more bifurcations than those seen in the fossils.

#### ﻿DT210

Another mine observed in the fossil *Eucalyptus* is very thin and threadlike, circumscribed by two secondary veins (DT210, Fig. 14A). Preserved length is 6.8 mm and width ranges between 0.1–0.2 mm. The mine has an elliptical oviposition site (see lowermost arrow in Fig. 14A), 0.3 mm in length and 0.1 mm in width. The mine follows a curvilinear path towards the leaf margin for 5.1 mm, with a modest width increase from 0.1 mm to 0.2 mm, before reaching the neck of the terminal chamber (see uppermost arrow in Fig. 14A). In turn, the terminal chamber is elliptical in shape, 1.4 mm in length and 0.4 mm in width. A thin, 0.05 mm thick reaction tissue is present throughout the mine. Frass is absent.

Four analogs for the thin, DT210 mines were observed in extant *Eucalyptus* specimens (Fig. 14B–E). Both fossil and extant mines share having a distinctive oviposition site (see arrows in Fig. 14), a path that is bounded by secondary veins (but see exceptions below), and an elliptical terminal chamber. The extant examples are, however, 4–5 times longer than the fossil mine, and the examples depicted in Fig. 14C, D are slightly thicker. In turn, the mine depicted in Fig. 14E has the oviposition site closer to the leaf margin and terminates beside the midvein, the opposite of what is seen in the fossil and the rest of the extant analog mines.

#### ﻿DT422

The last mine reported on the fossil *E. frenguelliana* corresponds to a linear mine occurring alongside the midvein, with minimal width increases throughout its course, assigned to DT422 (Fig. 15A). Preserved length is 12.5 mm and width ranges between 0.3–0.6 mm. The oviposition site is roughly triangular in shape, reaching 0.7 mm in height and 0.4 mm in width at the widest portion. The mine is directed towards the leaf base and follows the midvein throughout all its course, rapidly increasing in width from 0.4 mm to 0.6 mm during the first 0.4 mm of the mine’s length. This same width is retained throughout the remaining 9.3 mm of the mine’s course—although there are two slight constrictions that reduce the mine’s width to 0.3 mm—before reaching the neck of the terminal chamber (see arrow in Fig. 15A). The terminal chamber is elliptical in shape, 2.1 mm in length and 0.6 mm in width. Throughout the course of the mine, there is a distinct, 0.1–0.2 mm thick, carbonized reaction rim. Frass is absent.

Two extant analogs were observed for DT422. All three mines, whether fossil or extant, occur alongside the midvein and have a constant width that is maintained throughout the mine’s course, two very distinctive features unique to these mines. Furthermore, all mines share a path that is directed towards the base of the leaf, given that the elliptical terminal chambers (see arrows in Fig. 15) are found closest to the leaf base. Minimal differences between the fossil and extant damage are seen for the mine in Fig. 15B, wherein the upper layer of epidermis covering the mine is breached, likely due to environmental factors such as in vivo abrasion, which also affected the overall shape of the terminal chamber.

Mining insects attacking extant *Eucalyptus* species pertain to three orders, including Diptera, Hymenoptera, and Lepidoptera. For Diptera, the only recorded association is that of *Japanagromyza eucalypti* (Agromyzidae) mining *E. camaldulensis* Dehnh. leaves in New South Wales (Spencer 1963, 1977; Moore 1966), whereas three hymenopteran species pertaining to the genus *Phylacteophaga* (Pergidae) have been recorded mining several economically important *Eucalyptus* species (e.g., Moore 1966, 1972; Mayo et al. 1997). The lepidopteran fauna mining *Eucalyptus* is much more diverse, including several families such as Gracillariidae, Incurvariidae, Heliozelidae, and especially Nepticulidae (Moore 1966, 1972; Mazanec 1987; Common 1990; Hoare et al. 1997; Hoare and van Nieukerken 2013). However, mining lepidopterans associated with *Eucalyptus* are vastly understudied, and dozens of species remain to be described in families such as Gracillariidae, Heliozelidae, Incurvariidae, and Nepticulidae, among others (Common 1990).

### ﻿Pathogenic and oviposition traces

Pathogenic damage observed in the fossil *Eucalyptus* include necrotic tissue occurring along the leaf margin (DT114, Fig. 16A), covering 21.8 mm of leaf tissue and with a 0.2 mm thick reaction front; circular fungal blotches that display a prominent, carbonized core eccentrically positioned (DT261, Fig. 16C), with blotches measuring 0.5–4.7 mm in diameter, and internal carbonized cores that are 0.5–1.7 mm in diameter; as well as ovoidal blotches of necrotic tissue originating near the midvein (DT58, Fig. 17A), measuring 5.2 mm in length and 3 mm in width.

Oviposition traces include elliptical scars occurring throughout the leaf lamina (DT54, Fig. 17B, D), with individual scars measuring 0.5–2.1 mm in length and 0.2–0.7 mm in width; as well as narrow, lenticular lesions aligned lengthwise end-to-end in an arc paralleling the midvein (DT310, Fig. 17B, C), with individual scars measuring 2.1–2.6 mm in length and 0.6–1.7 mm in width. Reaction rims associated with these oviposition scars are 0.1 mm thick. These same lesions have been previously interpreted as odonatan endophytic oviposition scars (see fig. 2.3, 4 of Sarzetti et al. 2009 and fig. 1.1 of Romero-Lebrón et al. 2019) and were assigned to the ichnotaxa *Paleoovoidus arcuatum* (for the DT54 examples seen Fig. 17B, D), probably caused by members of the family Coenagrionidae; and *P. rectus* (for the DT310 example seen in Fig. 17B, C), probably induced by members of the family Lestidae (Sarzetti et al. 2009). The prior authors (Sarzetti et al. 2009; Romero-Lebrón et al. 2019) referred to the plant hosts by other names (“*Eucalyptus chubutensis*,” “*Myrcia chubutensis*”), but I clarify here that all are specimens of *Eucalyptus frenguelliana* (see Hermsen et al. 2012).

No extant analogs were found for the oviposition traces seen in the fossils. For pathogenic damage, an extant analog was observed for the necrotic tissue occurring along the leaf margin (DT114; Fig. 16A) in one *E. michaeliana* Blakely herbarium specimen (Fig. 16B). Both the fossil and extant damage coincide in terms of location, shape, and diffuse reaction tissue that is frequently deflected by secondary veins. In turn, circular fungal blotches with prominent, carbonized cores (DT261; Fig. 16C) were found across multiple extant *Eucalyptus* species (Fig. 16D–F), with very similar sizes, positions, and arrangement of the damage itself, wherein a diffuse reaction front contains a ring of seemingly unaltered leaf tissue that, in turn, contains an inner carbonized core. No extant analogs were found for DT58 (Fig. 17A).

## ﻿Discussion

By fully documenting insect and pathogenic damage on the fossil *E. frenguelliana* leaves from LH, accompanied by detailed morphological comparisons with damage on extant *Eucalyptus*, this study provides an important reference for ecological interactions through geologic time. The fossils preserve twelve types of external feeding, one of piercing-and-sucking, five of galls, and ten of mines. One gall trace has been re-designated as DT215 (previously treated as DT85 in Giraldo et al. 2025; see Methods), based on a helpful reviewer comment; this change does not affect the overall DT richness for the *E. frenguelliana* fossils. In addition, the description of three pathogenic DTs made here expands the known range of interactions on *E. frenguelliana* leaves, indicating that this plant host was not only used by a diverse array of herbivorous insects (Giraldo et al. 2025), but also by a broader group of organisms that accessed the leaf tissue through specialized structures such as fungal haustoria (Labandeira et al. 2007; Labandeira and Prevec 2014). Collectively, the full suite of 33 DTs identified in the fossil *E. frenguelliana* leaves indicate that it was an important ecological resource in the forests of ancient Patagonia.

Few morphological differences were seen when comparing the 28 herbivory-related DTs identified in the fossils with their corresponding analogs in extant *Eucalyptus* species (see Results). External feeding DTs, including hole feeding, margin feeding, surface feeding, and skeletonization were frequently encountered in extant *Eucalyptus* species (Figs 1–3), where they exhibit similar positions along the leaf lamina, overall shape, and thickness of the reaction tissue. For the circular scale insect covers assigned to DT77, part of the piercing-and-sucking FFG, two extant analogs were observed in an *E. notabilis* herbarium specimen (Fig. 4). Although the extant covers are bigger than their fossil counterparts, they all share the circular shape, waxy texture, and presence of concentric growth rings. In turn, the galling DTs observed in the fossils were frequently encountered in several extant *Eucalyptus* species (Fig. 5), with similar leaf deformation patterns (Fig. 5A, B), positioning along the midvein (Fig. 5C, D), length:width ratios and jagged reaction rims (Fig. 5E–I), as well as carbonized cores with masses of radiating tissue (Fig. 5J–L). For the highly diverse mining DTs, extant analogs were considerably scarcer (Figs 6–15; see Giraldo et al. 2025: table S1) when compared to the other FFGs. Extant analogs occur in as few as one *Eucalyptus* species (DT185 in *E. crebra* F.Muell.; Fig. 12) and in as many as seven (DT139; Fig. 10). Fossil and extant mines share several features, including total length, expansion pattern, oviposition site and shape, directionality of the mine, reaction tissue thickness, terminal chamber dimensions and shape, and (when present) frass deposition pattern.

Only two (DT114 and DT261; Fig. 16A, C) of the pathogenic traces observed in the fossils were documented in extant *Eucalyptus*, with similarities including size, distribution of the reaction fronts, and carbonized cores surrounded unaltered leaf tissue (Fig. 16). The remaining pathogenic trace (DT58; Fig. 17A) and the two oviposition scars (DT54 and DT310; Fig. 17B–D) identified in the fossils were not observed in extant *Eucalyptus* species, suggesting possible extinction, extirpation, or undersampling in herbarium sheets. However, the oviposition traces have been previously interpreted as odonatan oviposition lesions, probably made by members of the family Lestidae (for the DT54 seen in Fig. 17B, D) and Coenagrionidae (for the DT310 observed in Fig. 17B, C; Sarzetti et al. 2009; Romero-Lebrón et al. 2019).

As discussed by Giraldo et al. (2025), the nearly identical suites of shared leaf damage between fossil and extant *Eucalyptus* suggest that some of the insect herbivore lineages that fed on the ancient Patagonian gum trees tracked (and radiated on) their host genus through time and space. Host-tracking by insect herbivores has been previously observed in the fossil record, although most studies focus on one or very few associations (Opler 1973; Hickey and Hodges 1975; Labandeira et al. 1994; Wilf et al. 2000; Leckey and Smith 2015; Su et al. 2015; Adroit et al. 2020). Platanoid (Platanaceae) hosts have been mined by *Ectoedemia* (Nepticulidae) micromoths since the mid-Cretaceous of the western United States (Labandeira et al. 1994), and *Zingiberopsis* Hickey (Zingiberaceae) leaves from the latest Cretaceous and Eocene of the Western Interior of North America record stereotypical surface feeding traces made by beetles in the subfamily Hispinae (Chrysomelidae), an association that still occurs today in most of the Neotropical families in Zingiberales (Wilf et al. 2000). In turn, the leaf-blotch miner genus *Phyllocnistis* (Gracillariidae) has attacked *Cedrela* P.Browne (Meliaceae) leaves since at least the Eocene of Wyoming (Hickey and Hodges 1975), while wasps of the tribe Cynipini (Cynipidae) have induced galls in oak leaves since the Oligocene of the western United States (Leckey and Smith 2015). Finally, several mining Nepticulidae and Gracillariidae lineages have been associated with their oak hosts (*Quercus* L. spp.) since the Miocene of western North America (Opler 1973), and *Parrotia* C.A.Mey. (Hamamelidaceae) leaves from the Miocene of China and Pliocene of Germany record distinct curvilinear skeletonizations that are observed today in the same host-genus in Iran and China, probably produced by chrysomelid beetles belonging to the subfamilies Galerucinae or Alticinae (Adroit et al. 2020).

Studies comparing the full suite of associations observed in one plant host for millions of years and into the modern day are much scarcer. In Su et al. (2015), a total of twelve DTs were observed in oak leaves from the Pliocene of China, eleven of which were recognized in extant oaks in nearby forests. However, only two DTs are host-specialized mining interactions, and the remaining DTs are generalized external feeding associations that do not necessarily indicate the persistence of long-term associations (Su et al. 2015). In contrast, the work of Donovan et al. (2020, 2023) compared suites of insect herbivore and fungal damage associated with fossil *Agathis* Salisb. from Patagonia, derived from four latest Cretaceous to middle Eocene localities (including LH) spanning ca. 18 Myr, with that of extant *Agathis* species in the West Pacific. The highly similar damage traces between fossil and extant *Agathis*—including blotch mines, external foliage feeding, galls, possible armored scale insect (Diaspididae) covers, and a rust fungus (Puccinales)— suggest that the associated insect herbivore and fungal assemblages probably tracked the host genus through time and space (Donovan et al. 2020, 2023). However, most of the culprits responsible for the comparable extant damage remain unknown, and the same is true for *Eucalyptus* (see below), highlighting the importance of natural history observations and illustrations in reconciling fossil and extant interactions (Giraldo et al. 2025).

### ﻿Potential affinities of the insect culprits

External feeding DTs are frequently encountered today and in the fossil record (Labandeira et al. 2007), reflecting the high diversity and abundance of mandibulate insect herbivores (Carvalho et al. 2014). Orders such as Coleoptera, Phasmatida, and Lepidoptera contain hundreds of thousands of species that can create similarly shaped external feeding traces (Schoonhoven et al. 1998), especially when considering the multiple ontogenetic stages (larvae, nymphs, adults) that chew on leaves (Hochuli 2001). Moreover, external feeding DTs also include insect damage that is not caused by direct feeding, such as leaf cutting by ants and bees (Labandeira et al. 2007; Sarzetti et al. 2008). Due to this widespread mouthpart and behavioral convergence, few external feeding DTs observed in fossils have been assigned to specific taxonomic groups (but see Wilf et al. 2000; Adroit et al. 2020), and none of the extant or fossil external feeding traces described here (Figs 1–3) can be confidently assigned to an insect culprit lineage.

In contrast, most of the fossil DTs that have been taxonomically identified correspond to internal feeding such as leaf mining (e.g., Labandeira et al. 1994; Doorenweerd et al. 2015; Maccracken et al. 2021) and, more rarely, galling (e.g., Leckey and Smith 2015). Although most of the galls reported here (Fig. 5) have lost their three-dimensional structure during the fossilization process and cannot be reconciled with a particular gall-inducing group, some of the mines have morphological features consistent with the lepidopteran families Nepticulidae and Gracillariidae, as well as with Agromyzidae in Diptera. Although identifying insect culprit lineages from fossil mines is less precise than identification of adults because leaf mine characters have never been analyzed in a phylogenetic context, for many taxa there is a combination of characters that distinguishes the group (Labandeira et al. 2007; Winkler et al. 2010; Sohn et al. 2012; Doorenweerd et al. 2015). Relevant taxonomic characters include the shape and direction of the mine, shape and scar tissue of the oviposition site, frass deposition pattern, mine depth, and, very importantly, identity of the plant host (Labandeira et al. 2007; Doorenweerd et al. 2015)

Mines potentially affiliated with the pygmy moth family Nepticulidae include DT41 (Fig. 6) and DT92 (Fig. 8). Although leaf mine shape is highly variable in Nepticulidae, larvae generally create curvilinear galleries that avoid primary venation (as in the DT92 of Fig. 8A) or are only able to cross major veins during later instar stages (as in the DT41 of Fig. 6A; Doorenweerd et al. 2015). Nepticulid females oviposit on the leaf surface, and there is no scarring around the oviposition site (Sohn et al. 2012; Doorenweerd et al. 2015), a feature shared by the fossil DT41 and DT92 and their extant analogs (Figs 6, 8). Moreover, randomly distributed pellets filling the entire mine’s width are also consistent with Nepticulidae, despite frass deposition patterns being highly variable in the family (Doorenweerd et al. 2015). Most importantly, however, the affiliation to Nepticulidae is highly supported because the mine occurs on a *Eucalyptus* plant host. In fact, it is probable that the insects responsible for creating the fossil DT41 and DT92 mines on the Eocene *Eucalyptus* belong (or are related) to the nepticulid genus *Pectinivalva*, given close morphological similarities with mines created by pygmy moths pertaining to this genus (see figs. 117, 120, 123 in Hoare and van Nieukerken 2013; RJB Hoare, pers. comm.).

In turn, the fossil mine assigned to DT94 (Fig. 9A) is consistent with features of the highly diverse moth family Gracillariidae. Despite being commonly known as “leaf-blotch miners”, gracillariids also create linear-to-curvilinear mines in the sub-epidermal layer during the initial sap-feeding instars (Hering 1951; Labandeira et al. 1994; Hoare et al. 2019; Li et al. 2022), resulting in a distinctive brownish-to-silver hue in modern mines (Fig. 9B–D; Luo 2024). Although the silver color is not observable in the fossil, secondary veins are clearly crossing the mine’s path and the mine itself is discolored when compared to the rest of the leaf tissue, suggesting a sub-epidermal nature for the fossil mine (Fig. 9A). Moreover, some gracillariid larvae create short diversions in the mine’s path (see arrows in Fig. 9), cross the midvein near the apex where its thickness is at its minimum, extend down the other side of the leaf, and then construct a pupation chamber by using silk and contracting the sides of the leaf, resulting in a pinched appearance around the leaf margin (see arrows in Fig. 9; Hering 1951; Hoare et al. 2019; Luo 2024). All the abovementioned characters are readily visible in the DT94 fossil mine (Fig. 9A), supporting its affiliation to Gracillariidae.

The last mine that can be associated with an insect group corresponds to DT207 (Fig. 13A, B), which is probably affiliated with the fly family Agromyzidae. In a thorough revision of the Agromyzidae fossil record, Winkler et al. (2010) mentioned that leaf mines can be confidently diagnosed to this insect family by the presence of fluidized frass deposited in bands (or specks) that alternate between the two sides of the mine. Because dipteran leaf miners do not ingest cell walls, the resulting fluidized frass hardens to form dark bands on the mine’s interior, and given that agromyzid larvae lay on their sides and alternate between the sides of the mine to feed, the strips of frass often alternate in linear mines (Hering 1951; Winkler et al. 2010). Both features are observed in the DT207 fossil mines (Fig. 17A, B), supporting their affiliation to Agromyzidae.

### ﻿Eucalypt fossil foliage and associated leaf damage

**Figure 18.**
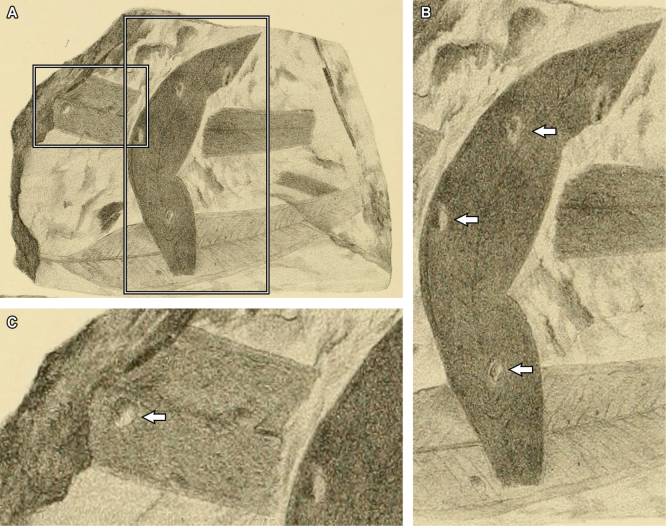
Putative *Eucalyptus* fossils from the Pliocene of Victoria, Australia, illustrated by McCoy (1876) (see also Hill 1994) and reproduced here under Australian Public Domain. A. Illustration of a rock slab with multiple leaf remains depicted in fig. 1 (Plate XXXIX) of McCoy (1876); B. Close-up of a leaf in (A), arrows indicate galls that could be assigned to DT32 and DT33, with similar leaf deformation patterns to those observed in *E. frenguelliana* from Laguna del Hunco (Fig. 2D); C. Close-up of a leaf in (A), arrow marks a gall probably corresponding to DT33.

Besides the *E. frenguelliana* leaf material studied here, other fossil foliage referred to eucalypts has been described from the Cenozoic of Australia and New Zealand (McCoy 1876; Ettingshausen 1895; Maiden 1924; Holmes et al. 1982; Pole 1993, 1994, 2019; Pole et al. 1993). Although none of these studies has explicitly reported the presence of insect or pathogenic damage, some of the illustrated material has galling traces. Putative *Eucalyptus* leaves from the early Miocene of New Zealand have galls occurring alongside primary veins that could be assigned to DT33 (fig. 4A, D in Pole 1993). Indistinct galling traces on the leaf lamina (DT32) and alongside primary veins (DT33), as well as elongated galls on the midvein (DT85), are observed on leaves referred to eucalypts from the early Eocene of Queensland (figs. 16, 17 in Pole 2019). Similarly, putative *Eucalyptus* leaves from the Oxley Basin of Queensland and the middle Miocene of New South Wales have indistinct galls occurring along the leaf lamina assignable to DT32 (fig. 10 in Plate IV of Ettingshausen 1895 and fig. 3D in Holmes et al. 1982). However, the most accurate depiction of leaf galling is an illustration of a rock slab from the Pliocene of Daylesford (Victoria), featuring several leaves of the fossil species *Eucalyptus pluti* McCoy (McCoy 1876). These *E. pluti* leaves, considered a genuine record of *Eucalyptus* by Hill (1994), exhibit numerous galls on the leaf lamina (DT32) and alongside primary veins (DT33), which appear to deform the original shape of the leaf (Fig. 18), a pattern noted for some of the galling traces on the *E. frenguelliana* leaves from LH (Fig. 5A). In trying to depict the fossil specimens as accurately as possible, as was typical of the earlier scientific literature that primarily focused on natural history observations, McCoy (1876) was probably the first person to document galls on fossil eucalypts.

## ﻿Conclusions

The detailed documentation of the leaf damage associated with *E. frenguelliana* fossils from the early Eocene Laguna del Hunco locality in Patagonia shows that this plant host was used by a wide array of insects and pathogens. The *E. frenguelliana* leaves record a diverse suite of damage, including external feeding traces, scale insect covers, galls, mines, pathogenic marks, and oviposition scars. Most of these damage traces were also observed in extant *Eucalyptus* specimens, indicating that some of the ancient insect lineages tracked their plant host genus through time and space. However, except for three fossil mines that are probably affiliated with the moth families Nepticulidae and Gracillariidae, as well as one fossil mine consistent with Agromyzidae, most of the damage was produced by unknown culprits, highlighting gaps in our knowledge of *Eucalyptus* ecological associates and their assembly through evolutionary time.

## ﻿Additional information

### Use of AI

No use of AI was reported.

### Author ORCIDs

L. Alejandro Giraldo https://orcid.org/0000-0001-9824-5521

### Data availability

All of the data that support the findings of this study are available in the main text or Supplementary Information.

## Supplementary materials

Supplementary material 1 Catalog of fossil specimens Data type xlsx https://binary.pensoft.net/file/1467257This dataset is made available under the Open Database License (http://opendatacommons.org/licenses/odbl/1.0/). The Open Database License (ODbL) is a license agreement intended to allow users to freely share, modify, and use this Dataset while maintaining this same freedom for others, provided that the original source and author(s) are credited.L. Alejandro Giraldo
